# Supplementary material for: Modeling glioblastoma heterogeneity as a dynamic network of cell states
Source: Mol Syst Biol. 2021 Sep 16;17(9):e10105. doi: 10.15252/msb.202010105 (PMC8444284; doi:10.15252/msb.202010105)
Supplement: Supplementary file 6 — Source Data for Figure 5 [file MSB-17-e10105-s004.zip › Figure5A_sourcedata/GSEA_3017/hallmarks_stateE.GseaPreranked.1621934677158/HALLMARK_G2M_CHECKPOINT.html]

Details for gene set HALLMARK\_G2M\_CHECKPOINT[GSEA]

|  || Dataset | state63017 |
| Phenotype | NoPhenotypeAvailable |
| Upregulated in class | na\_neg |
| GeneSet | HALLMARK\_G2M\_CHECKPOINT |
| Enrichment Score (ES) | -0.6063061 |
| Normalized Enrichment Score (NES) | -3.1910045 |
| Nominal p-value | 0.0 |
| FDR q-value | 0.0 |
| FWER p-Value | 0.0 |
Table: GSEA Results Summary

  

Fig 1: Enrichment plot: HALLMARK\_G2M\_CHECKPOINT      
 Profile of the Running ES Score & Positions of GeneSet Members on the Rank Ordered List

  

| PROBE | GENE SYMBOL | GENE\_TITLE | RANK IN GENE LIST | RANK METRIC SCORE | RUNNING ES | CORE ENRICHMENT || 1 | SQLE |  |  | 168 | 0.319 | -0.1928 | No |
| 2 | TNPO2 |  |  | 273 | -0.251 | -0.3112 | No |
| 3 | HMGA1 |  |  | 313 | -0.261 | -0.3528 | No |
| 4 | MCM5 |  |  | 337 | -0.265 | -0.3754 | No |
| 5 | FOXN3 |  |  | 339 | -0.266 | -0.3722 | No |
| 6 | STAG1 |  |  | 360 | -0.271 | -0.3912 | No |
| 7 | CDC25A |  |  | 368 | -0.273 | -0.3948 | No |
| 8 | SLC12A2 |  |  | 389 | -0.279 | -0.4137 | No |
| 9 | HSPA8 |  |  | 392 | -0.280 | -0.4114 | No |
| 10 | MTF2 |  |  | 405 | -0.284 | -0.4208 | No |
| 11 | NASP |  |  | 410 | -0.285 | -0.4207 | No |
| 12 | JPT1 |  |  | 439 | -0.296 | -0.4488 | No |
| 13 | CTCF |  |  | 443 | -0.297 | -0.4473 | No |
| 14 | KPNB1 |  |  | 445 | -0.298 | -0.4435 | No |
| 15 | KMT5A |  |  | 448 | -0.298 | -0.4408 | No |
| 16 | CDC7 |  |  | 451 | -0.299 | -0.4382 | No |
| 17 | HNRNPD |  |  | 456 | -0.301 | -0.4378 | No |
| 18 | SMC1A |  |  | 472 | -0.307 | -0.4503 | No |
| 19 | NUMA1 |  |  | 477 | -0.307 | -0.4499 | No |
| 20 | BARD1 |  |  | 510 | -0.318 | -0.4823 | No |
| 21 | POLA2 |  |  | 521 | -0.320 | -0.4887 | No |
| 22 | PLK4 |  |  | 551 | -0.334 | -0.5173 | No |
| 23 | SAP30 |  |  | 558 | -0.339 | -0.5187 | No |
| 24 | MCM3 |  |  | 560 | -0.340 | -0.5141 | No |
| 25 | TROAP |  |  | 562 | -0.341 | -0.5096 | No |
| 26 | HMGB3 |  |  | 577 | -0.348 | -0.5203 | No |
| 27 | DDX39A |  |  | 590 | -0.353 | -0.5285 | No |
| 28 | PRIM2 |  |  | 607 | -0.362 | -0.5413 | No |
| 29 | NEK2 |  |  | 628 | -0.374 | -0.5586 | No |
| 30 | POLE |  |  | 652 | -0.395 | -0.5791 | No |
| 31 | GINS2 |  |  | 658 | -0.399 | -0.5782 | No |
| 32 | NUP50 |  |  | 677 | -0.415 | -0.5925 | No |
| 33 | RPA2 |  |  | 688 | -0.424 | -0.5972 | No |
| 34 | STIL |  |  | 692 | -0.425 | -0.5935 | No |
| 35 | NSD2 |  |  | 693 | -0.426 | -0.5864 | No |
| 36 | E2F3 |  |  | 700 | -0.431 | -0.5862 | No |
| 37 | CHEK1 |  |  | 704 | -0.433 | -0.5825 | No |
| 38 | E2F1 |  |  | 708 | -0.435 | -0.5787 | No |
| 39 | TTK |  |  | 724 | -0.454 | -0.5888 | No |
| 40 | POLQ |  |  | 726 | -0.455 | -0.5823 | No |
| 41 | RAD21 |  |  | 728 | -0.458 | -0.5758 | No |
| 42 | CCNF |  |  | 732 | -0.460 | -0.5716 | No |
| 43 | LBR |  |  | 739 | -0.463 | -0.5709 | No |
| 44 | KIF5B |  |  | 770 | -0.515 | -0.5977 | Yes |
| 45 | CDC45 |  |  | 773 | -0.521 | -0.5913 | Yes |
| 46 | DTYMK |  |  | 774 | -0.522 | -0.5825 | Yes |
| 47 | STMN1 |  |  | 778 | -0.529 | -0.5771 | Yes |
| 48 | CENPA |  |  | 782 | -0.538 | -0.5716 | Yes |
| 49 | INCENP |  |  | 789 | -0.558 | -0.5693 | Yes |
| 50 | CDC20 |  |  | 793 | -0.562 | -0.5634 | Yes |
| 51 | HMGN2 |  |  | 795 | -0.564 | -0.5551 | Yes |
| 52 | RACGAP1 |  |  | 796 | -0.566 | -0.5456 | Yes |
| 53 | CDC6 |  |  | 807 | -0.584 | -0.5476 | Yes |
| 54 | PLK1 |  |  | 809 | -0.586 | -0.5389 | Yes |
| 55 | CHAF1A |  |  | 810 | -0.588 | -0.5291 | Yes |
| 56 | LMNB1 |  |  | 818 | -0.606 | -0.5271 | Yes |
| 57 | AURKA |  |  | 824 | -0.623 | -0.5226 | Yes |
| 58 | AURKB |  |  | 829 | -0.634 | -0.5166 | Yes |
| 59 | BUB3 |  |  | 834 | -0.641 | -0.5106 | Yes |
| 60 | CDKN3 |  |  | 836 | -0.653 | -0.5008 | Yes |
| 61 | BUB1 |  |  | 837 | -0.653 | -0.4898 | Yes |
| 62 | KIF22 |  |  | 842 | -0.678 | -0.4831 | Yes |
| 63 | BIRC5 |  |  | 843 | -0.679 | -0.4717 | Yes |
| 64 | BRCA2 |  |  | 850 | -0.694 | -0.4672 | Yes |
| 65 | KIF2C |  |  | 855 | -0.721 | -0.4598 | Yes |
| 66 | KIF4A |  |  | 861 | -0.736 | -0.4533 | Yes |
| 67 | HMMR |  |  | 863 | -0.740 | -0.4421 | Yes |
| 68 | FBXO5 |  |  | 864 | -0.742 | -0.4296 | Yes |
| 69 | CCNB2 |  |  | 865 | -0.745 | -0.4171 | Yes |
| 70 | NDC80 |  |  | 869 | -0.762 | -0.4078 | Yes |
| 71 | ORC6 |  |  | 870 | -0.766 | -0.3949 | Yes |
| 72 | CCNA2 |  |  | 872 | -0.770 | -0.3832 | Yes |
| 73 | KIF23 |  |  | 873 | -0.772 | -0.3702 | Yes |
| 74 | TMPO |  |  | 875 | -0.778 | -0.3583 | Yes |
| 75 | KIF20B |  |  | 876 | -0.779 | -0.3452 | Yes |
| 76 | PTTG1 |  |  | 880 | -0.791 | -0.3355 | Yes |
| 77 | EZH2 |  |  | 886 | -0.810 | -0.3278 | Yes |
| 78 | UBE2S |  |  | 887 | -0.815 | -0.3141 | Yes |
| 79 | KIF15 |  |  | 890 | -0.834 | -0.3024 | Yes |
| 80 | KPNA2 |  |  | 895 | -0.858 | -0.2927 | Yes |
| 81 | DBF4 |  |  | 896 | -0.864 | -0.2782 | Yes |
| 82 | SMC2 |  |  | 897 | -0.867 | -0.2636 | Yes |
| 83 | CENPE |  |  | 899 | -0.873 | -0.2501 | Yes |
| 84 | PBK |  |  | 900 | -0.880 | -0.2354 | Yes |
| 85 | SMC4 |  |  | 902 | -0.886 | -0.2217 | Yes |
| 86 | CKS1B |  |  | 909 | -0.912 | -0.2134 | Yes |
| 87 | CKS2 |  |  | 911 | -0.922 | -0.1991 | Yes |
| 88 | KIF11 |  |  | 913 | -0.957 | -0.1842 | Yes |
| 89 | MYBL2 |  |  | 914 | -0.959 | -0.1681 | Yes |
| 90 | KNL1 |  |  | 915 | -0.963 | -0.1519 | Yes |
| 91 | PRC1 |  |  | 919 | -0.980 | -0.1390 | Yes |
| 92 | MAD2L1 |  |  | 921 | -0.990 | -0.1235 | Yes |
| 93 | UBE2C |  |  | 928 | -1.108 | -0.1120 | Yes |
| 94 | NUSAP1 |  |  | 929 | -1.112 | -0.0933 | Yes |
| 95 | TPX2 |  |  | 930 | -1.124 | -0.0744 | Yes |
| 96 | CENPF |  |  | 935 | -1.200 | -0.0590 | Yes |
| 97 | CDK1 |  |  | 938 | -1.248 | -0.0404 | Yes |
| 98 | MKI67 |  |  | 941 | -1.407 | -0.0191 | Yes |
| 99 | TOP2A |  |  | 942 | -1.417 | 0.0047 | Yes |
Table: GSEA details [plain text format]

  

Fig 2: HALLMARK\_G2M\_CHECKPOINT: Random ES distribution      
 Gene set null distribution of ES for **HALLMARK\_G2M\_CHECKPOINT**
